# Shortened High-dose Palliative Radiotherapy for Lung Cancer (SHiP-Rt): protocol for a single-arm, multicentre, phase II study

**DOI:** 10.1136/bmjopen-2025-111350

**Published:** 2026-02-02

**Authors:** Raj Kumar Shrimali, Ellanna Griffin, Vicky Sturgess, Matthew Jones, Louise Hiller, Jane Rogers, Joanna Hamilton, Charles Peebles, Bleddyn Jones, Janet Dunn

**Affiliations:** 1 Institute for Precision Diagnostic & Translational Medicine, University Hospitals Coventry and Warwickshire NHS Trust, Coventry, UK; 2 University of Warwick Medical School, Coventry, UK; 3 University Hospitals Coventry and Warwickshire NHS Trust, Coventry, UK; 4 University of Warwick Warwick Clinical Trials Unit, Coventry, UK; 5 University Hospitals Birmingham NHS Foundation Trust, Birmingham, UK; 6 Patient and Public Research Advisory Group, University Hospitals Coventry and Warwickshire NHS Trust, Coventry, UK; 7 Oxford Institute for Radiation Oncology, Oxford, UK

**Keywords:** Respiratory tract tumours, RADIOTHERAPY, Lung Neoplasms, Radiation oncology

## Abstract

**Introduction:**

Significant advances in systemic therapy have improved survival for patients with advanced-stage non-small cell lung cancer (NSCLC). However, the present treatment strategies and dose-fractionation for high-dose palliative radiotherapy (RT) are based on trials from the 1990s, when RT planning was simple with less precise delivery. Contemporary lung RT uses 4D-CT, volumetric modulated arc radiotherapy, aided by online verification using cone beam CT, which enables greater accuracy and better target volume coverage, while reducing doses to normal organs at risk. The Shortened High-dose Palliative Radiotherapy for Lung Cancer study aims to evaluate the safety and feasibility of reducing the number of RT fractions and RT duration, using contemporary planning, verification and delivery techniques.

**Methods and analysis:**

This single-arm, multicentre, phase-II study will test the shortened hypofractionated accelerated palliative RT regimen of 30 Gy in 6 alternate-day fractions, with strict normal tissue dose constraints. We aim to recruit 37 patients across 4 sites within the West Midlands. Quality assurance for the RT is supported by the Radiotherapy Trials Quality Assurance Group (RTTQA). Patients with locally advanced or metastatic NSCLC, who are candidates for high-dose palliative RT, before or after first-line systemic therapy, are eligible for recruitment. The primary objective of this study is to assess the safety of the proposed dose-fractionation. Secondary objectives include evaluating toxicity profiles, patient-reported outcome measures, time to progression, feasibility and the National Health Service cost-saving.

**Ethics and dissemination:**

This study is conducted in accordance with the International Council for Harmonisation Good Clinical Practice (ICH GCP) guidelines and all applicable regulatory frameworks, including, but not limited to, the UK policy framework for health and social care research, as well as the Health Research Authority and Health and Care Research Wales regulations. Approval for the study was granted on 18 April 2024 (IRAS project ID: 332998; REC reference: 24/WM/0032). The chief investigator is responsible for obtaining informed consent from participants. Any individual delegated this responsibility is thoroughly authorised, trained and competent to conduct the informed consent process. On completion of the trial, the results will be shared with participants in a plain language summary and will be submitted for publication in a peer-reviewed journal. If successful, this study will inform a phase III randomised controlled trial to assess efficacy. For updates on the study, visit the study web page (https://research.mededcoventry.org/About-Us/Meet-The-Team/TMU/Ship-Rt).

**Trial registration number:**

NCT06483308.

**Strengths and limitations of this study**

- Inclusion and exclusion criteria were peer reviewed by patient and public involvement groups and the National Cancer Research Institute (NCRI) lung-Clinical Studies Group and tightened to make them relevant to the real-world patient population, allowing for rigorous hypothesis testing in a representative sample population.
- The primary endpoint is valid, reproducible, relevant to the target population and responsive to the intervention being studied.
- Simons’s two-stage design is a simple, ethical and efficient adaptive design, with planned interim analysis and clear criteria for stopping or progressing to the next stage; this design is best suited to our phase II oncology trial, where the primary endpoint for each participant is obtained within 4 weeks of their completing radiotherapy.
- The patient population is prone to high rates of patient loss or attrition from disease progression or death; therefore, our sample size allows for a 15% drop-out rate.

## Introduction

 Non-small cell lung cancer (NSCLC) is a leading cause of cancer-related deaths in the UK and worldwide.^1^,^4^ About 48 000 patients in the UK were diagnosed with lung cancer in 2017. About half of all patients with NSCLC present with metastatic disease.^3^,^5^

Treatment for stage IV NSCLC is aimed at maintaining or improving quality of life by controlling symptoms and prolonging survival. These patients are not curable due to the presence of metastases^6^ and have a 1-year survival of under 20% and a 5-year survival of about 3%.^4^ Advances in systemic therapy have resulted in survival improvement,^7^,^13^ leading to significant expansion in use of targeted therapy using tyrosine kinase inhibitors, immunotherapy, chemotherapy and combination treatments.^13^ Radiotherapy (RT) is used for about 30% of patients with stage IV NSCLC, for local control and symptomatic metastases, either at diagnosis or on disease progression after systemic therapy.^5^ Palliative thoracic RT is also used for patients who are unresponsive to systemic therapy or patients unsuitable for systemic therapy.^14^ High-dose palliative radiotherapy using 30–39 Gy in 10–13 fractions is recommended by the Royal College of Radiologists (2020) for patients with Eastern Cooperative Oncology Group Performance Status 0–2 (online supplemental appendix 4) who are likely suitable for multiple lines of systemic therapy.^15^ The most widely used regimen across the UK is 36 Gy in 12 fractions over 16 days, supported by randomised controlled trial (RCT) evidence from the 1990s, when radiotherapy techniques were simple and less precise.^16^

Clinically relevant organs at risk for palliative thoracic RT are the oesophagus, normal lung tissue and spinal cord. Within the Cochrane review (2015), radiation-related oesophagitis was reported in 8 out of 14 studies (1301 patients) either as patient-reported or physician-assessed toxicity.^17^ The mean rate of grade 3–4 oesophagitis was reported as 25.7% in the intervention groups (that used more fractions) and 22.3% within the control groups (using fewer fractions). Radiation pneumonitis was graded or reported in only 3 out of 14 trials (533 patients) included in the meta-analysis, with the mean reporting rate of 3.9% in the control groups and 2.4% in the intervention group.^17^ The spinal cord remains an organ of concern because of the rare but serious late complication of radiation myelopathy (RM), that was reported by the Medical Research Council Lung Cancer Working Party (MRC-LCWP) in 1996.^18^ The pooled data on 1048 patients from three RCTs conducted by the MRC-LCWP was analysed, and RM was reported in five patients treated with either 17 Gy in 2 fractions (1 week apart) or 39 Gy in 13 fractions (5 fractions/week).^19^ The median survival after palliative RT ranges from 6.2 to 9 months^20^ and 1-year survival was reported as 39%.^17 18^ More recently, 30-day mortality rates have been reported as 9% in a large retrospective single-centre study and about 14% in a large population-based study.^20 21^

**Figure 1:**
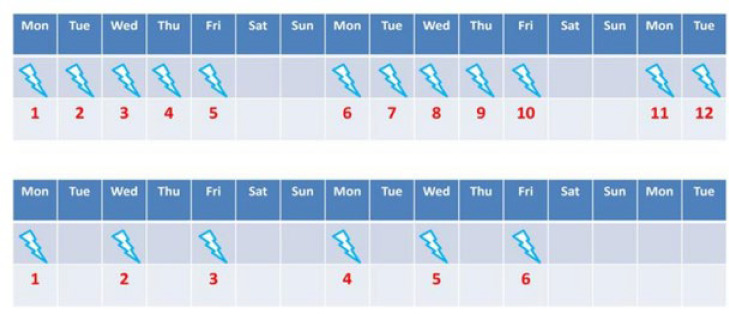
A diagram to depict the differences between the standard of care (top row) and the study regimen (bottom row).

The aim of the present study is to investigate the safety and feasibility of reducing the number of rt fractions and the treatment duration, compared with the current standard of care (36 Gy in 12 fractions over 16 days), by using 30 Gy in 6 fractions over 12 days (figure 1), provided the well-defined dose and biologically effective dose (BED) constraints are achievable.

## Methods and analysis

The proposed regimen of 30 Gy in 6 alternate-day fractions is comparable to the current standard treatment for tumour control, with similar BED and equivalent dose in 2 Gy fractions.^22^,^25^ From established models for effects of radiation, the proposed regimen should have similar efficacy for tumour control, although this needs to be tested in a phase III RCT, once the regimen is shown to be safe and feasible.^22^,^24^ High-dose palliative RT should be short and sharp and enable appropriate patients to move promptly onto definitive systemic therapy. A 12-fraction, 16-day radiotherapy regimen seems inappropriate when 9%–14% of patients die within 30 days and survival is limited.^20^ With advances in RT planning, verification and delivery techniques, it may be possible to safely treat patients with an adequate dose of palliative RT in fewer fractions, as demonstrated in a recent planning study.^25^ It is possible to keep doses to normal tissues and organs within tolerance by carefully adhering to the established dose constraints during planning and robust verification before delivery.

### Objectives

#### Primary objective

To assess the safety of reducing the number of radiotherapy fractions to 30 Gy in 6 alternate-day fractions over 12 days from the current standard of care by using advanced radiotherapy planning, verification and delivery techniques.

*Hypothesis:* With advances in RT planning, verification and delivery techniques, it is possible to safely treat patients with an adequate dose of palliative radiation in fewer fractions. It is possible to keep doses to normal tissues and organs within tolerance by carefully adhering to the established dose constraints during planning and robust verification before delivery.

#### Secondary objectives

- To monitor the overall toxicity profile, including cough, dyspnoea and pneumonitis using CTCAE version 4.03.
- To assess the feasibility of recruiting patients and treating them in a timely manner while maintaining necessary gaps and washout periods.
- To assess the time to progression (TTP).
- To explore the trend with patient-reported outcome measures (PROMS) for quality of life.
- To assess the cost savings from the proposed treatment.
- To assess the proposed treatment acceptability to patients and the feasibility to carry it out.

### Patient and public involvement

Through the Patient and Public Research Advisory Group at the UHCW Coventry, we presented the research idea and proposal to a group of patient representatives. Their feedback informed the study’s design and acceptability. Subsequently, we have appointed a patient representative to the study management group. Formal input was obtained from this patient representative and used for all patient-facing literature. They will stay on the trial management group (TMG) for the duration of this study and will help monitor the conduct and progress of this study. Their involvement has been valuable in establishing the database, choice of data fields and outcomes collected, in policies concerning the database, analysis of data and in the proposed dissemination of results.

### Trial design and setting

Shortened High-dose Palliative Radiotherapy for Lung Cancer (SHiP-Rt) is a phase II prospective, single-arm study with a Simon two-stage optimal design (including safety and feasibility). This is a multicentre study, managed by the TMU at UHCW Coventry. The other recruiting sites are University Hospitals Birmingham NHS Trust and The Shrewsbury and Telford Hospital NHS Trust. University Hospitals of North Midlands NHS Trust is waiting to open. A comprehensive table of roles and responsibilities of the individuals involved is included in online supplemental file. Participating centres have access to contemporary radiotherapy techniques as described in the Radiotherapy Protocol (online supplemental file). All participating sites are recruiting, treating and continuing care for study patients as per the protocol and contemporary guidelines. Professionals looking after patients recruited within this study are working within their scope of practice. Participant populations are identified from either the multi-disciplinary team (MDT) or the oncology clinic.

### Eligibility criteria

Patients with locally advanced or metastatic NSCLC, where high-dose palliative radiotherapy (typically 36 Gy in 12 fractions) is being considered for local control or symptomatic metastases, either upfront prior to systemic therapy, or on disease progression after systemic therapy. Those identified at the respective MDT meetings and oncology clinics, after histological diagnosis and staging investigations are complete.

#### Inclusion criteria

All of the following inclusion criteria must be satisfied:

- Age ≥18.
- Patient has locally advanced (stage IIIB or IIIC) or metastatic (stage IV) NSCLC.
- Patient is treatment naïve or had limited progression after first-line systemic therapy (using chemotherapy, immunotherapy or targeted therapy).
- Patient is suitable for high-dose palliative RT (36 Gy in 12 fractions or 39 Gy in 13 fractions).
- Obtained written informed consent for the SHiP-Rt study.
- Patients receiving RT after first-line systemic therapy must have a wash-out period of at least 3 weeks (ie, 3–4 weeks).
- Treatment-naïve patients should be able to proceed to definitive systemic therapy without undue delay, that is, within 3–4 weeks.

#### Exclusion criteria

The presence of any of the following criteria would render the patient ineligible for this study:

- Contraindication for thoracic RT.
- Requiring lung RT after second-line systemic therapy for NSCLC.
- Has more than one cancer that requires active treatment.
- On cytotoxic treatment for rheumatoid arthritis or connective tissue disorders.
- Poor life expectancy, likely less than 6 months.
- Patients with difficulty regarding compliance with the study treatment or follow-up.
- Previous radiotherapy to the same area.

### Outcomes

#### Primary outcome

The proportion of patients successfully completing treatment without reporting grade 3–4 oesophagitis, within 4 weeks of completing RT (using CTCAE version 4.03), out of all patients starting treatment in this study. The time points at which the toxicity will be assessed and recorded within 12 months of completion of radiotherapy are described in the schedule of events.

#### Secondary outcomes

- Toxicity assessments for common acute and late toxicity using CTCAE version 4.03.
- Assess the feasibility of recruiting patients (number of patients screened, eligible, recruited and withdrawn) and treating them in a timely manner while maintaining necessary gaps and washout periods. Record the time at which systemic therapy was initiated from recruitment and from the end of radiotherapy.
- TTP—documentation of the time at which disease progression occurs from the end of radiotherapy.
- PROMS responses using EORTC QLQ-C30 and QLQ-LC13 at baseline and timepoints as described in the schedule of events.
- Calculations of cost savings from the proposed treatment regimen.
- The acceptability of the treatment to participants by recording how many participants participate in the study versus how many consent versus how many complete treatment.

### Sample size

The primary endpoint is the proportion of patients successfully completing treatment without reporting grade 3–4 oesophagitis, using CTCAE version 4.03 out of all patients starting treatment within this study.

The study regimen will be considered as:

- Feasible, if >70% patients complete treatment and do NOT report grade 3–4 oesophagitis.
- Not feasible, if <45% patients complete treatment and do NOT report grade 3–4 oesophagitis.

Simon’s two-stage design (Simon, 1989) ^26^ has been adopted. The null hypothesis that the true response rate is 45% will be tested against a one-sided alternative. Recruiting 37 patients will allow detection of the rate being ≤45% or ≥70%, with one-sided 5% significance and 85% power. This calculated sample size allows for a 15% attrition rate.

#### Stage 1 analysis

Patients will be accrued until 11 patients have primary endpoint information available. If there are five or fewer patients who completed treatment and did NOT report grade 3–4 oesophagitis (ie, six or more who either failed to complete treatment, or reported grade 3–4 oesophagitis), the study will be stopped early for futility. Otherwise, it will continue to recruit.

#### Stage 2 analysis

Additional patients will be accrued until a minimum of 31 patients have primary endpoint information available is reached (anticipated to be attained with 37 patients recruited). If 19 patients or more completed treatment and did NOT report grade 3–4 oesophagitis, the null hypothesis will be rejected, and the regimen will be considered feasible.

### Recruitment

**Figure 2:**
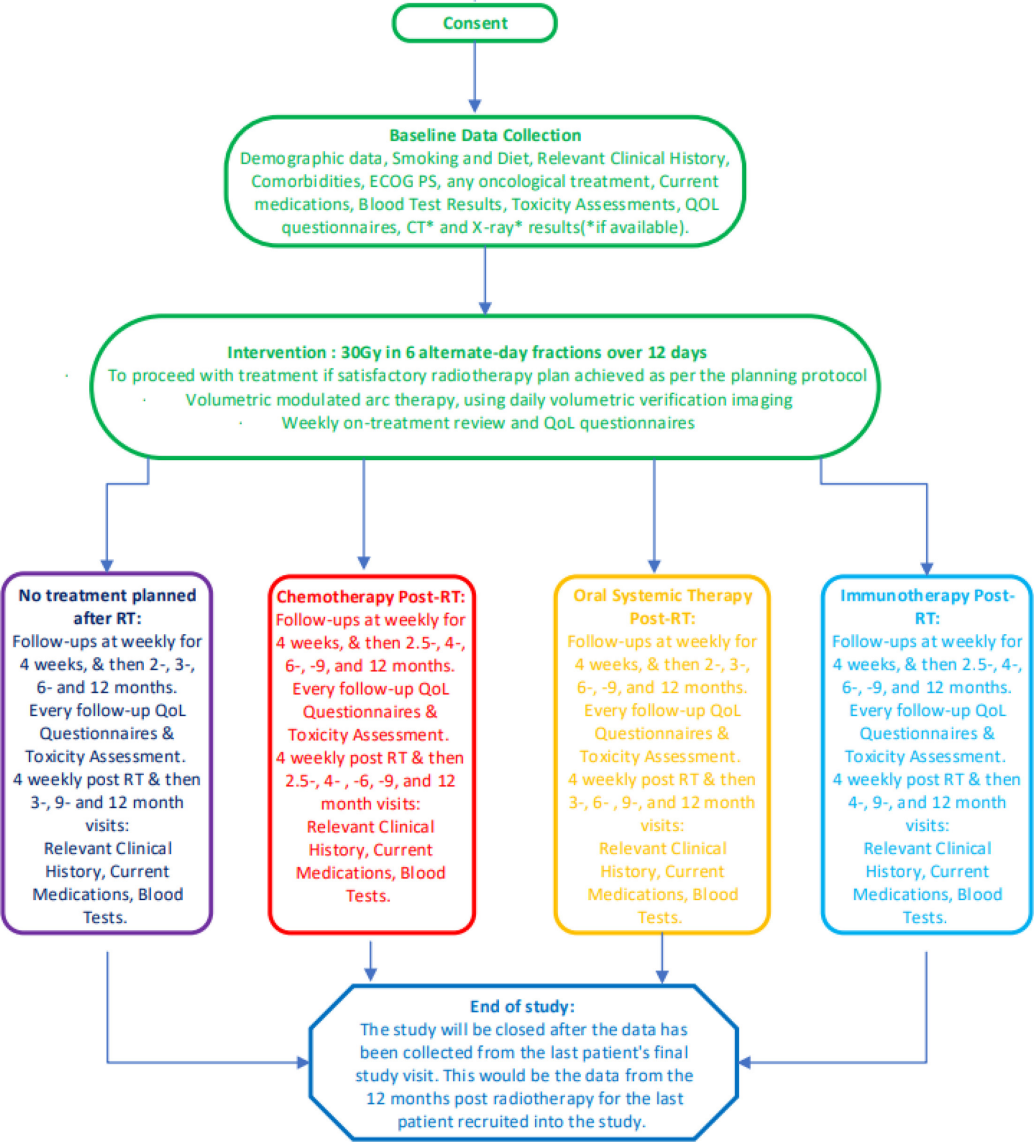
Flow of participants through the study. ECOG PS, Eastern Cooperative Oncology Group Performance Status; QoL, quality of life; RT, radiotherapy.

The patients are identified in the lung cancer pathway at the time of referral to the oncology service, in the MDT meetings or in an oncology clinic. They are screened at the outpatient clinic appointment and offered the Participant Information Sheet (PIS) (online supplemental appendix 5). They are consented at the time of booking the radiotherapy or at the subsequent clinic appointment or the planning CT appointment using the consent form as shown in online supplemental appendix 6. A study flow diagram is shown in figure 2. Information is collected regarding participants who are screened and for participants who are not recruited into the study, or those who decline the study, where data are being collated for Consolidated Standards of Reporting Trials or other similar reasons for reporting the generalisability of the results.

**Figure 3:**
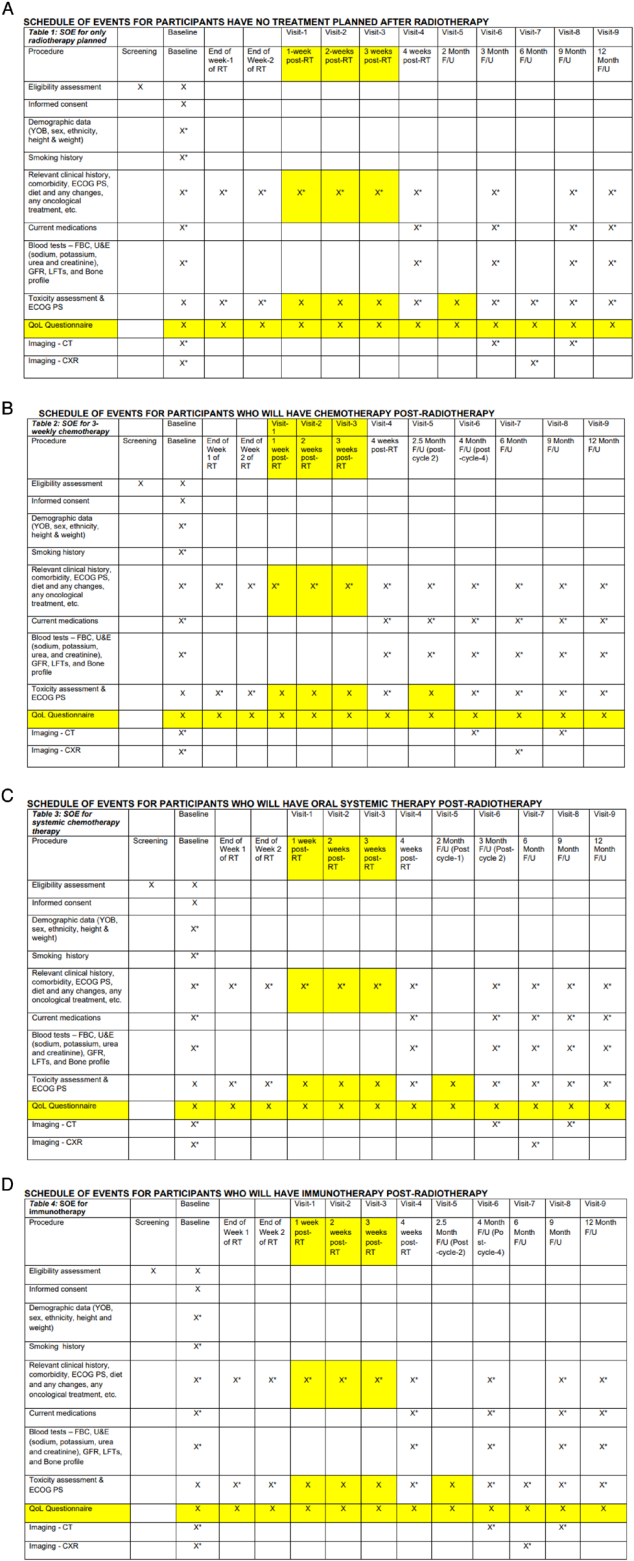
(A) Schedule of events—for patients who have no treatment planned after radiotherapy. (B) Schedule of events—for patients who will have chemotherapy after radiotherapy. (C) Schedule of events—for patients who will have oral systemic therapy after radiotherapy. (D) Schedule of events—for patients who will have immunotherapy after radiotherapy. X* - Standard of Care. The yellow highlighted boxes are procedures that are outside of the standard of care. The times at which the participant is followed-up depending on whether or not the participant has further treatment post-radiotherapy. These are not exact timepoints but a guideline for procedures. Visits 1-3 must be completed within +/- 3 days and from visits 4-9 must be completed within +/- 1 week. CXR (Chest X-ray) ; ECOG PS (Eastern Cooperative Oncology Group Performance Status); FBC, (full blood count); F/U (follow-up); GFR (glomerular filtration rate); LFT (liver function tests); QoL (quality of life); RT (radiotherapy); SOE (schedule of events); U&E (urea and electrolytes); YOB (year of birth).

Once entered into the study, the schedule of follow-up events does vary according to the further treatment plan for the patients. This is highlighted in figure 3.

### Data collection

The source documentation includes electronic hospital records and treatment databases, including radiotherapy planning and treatment databases. The necessary data are collected and transcribed onto a case report form (CRF). It is an electronic document (eCRF). The CRF data are used to perform the statistical analysis for the study. As this is a multicentre study, we are carrying out direct electronic entry and automatic upload into the database, for speed efficiency and accuracy.

- The data are collected by co-investigators and other authorised personnel listed in the delegation log.
- The types of data that are collected include patient demographics, tumour characteristics, details of treatment including radiotherapy dose-volume parameters, toxicity data and follow-up data.

### Data management

On confirmation of eligibility, participants are assigned a unique ID number, which is used to identify all documents associated with that participant for the duration of the study. All data, including source documents and eCRFs, is stored exclusively on hospital computer systems that are password-protected and access to these is defined and controlled by those on the delegation log. Direct access is granted to authorised representatives from the Sponsor, host institution and the regulatory authorities to permit study-related monitoring, audits and inspections—in line with participant consent. Following the resolution of queries and confirmation of study close-out by the chief investigator, all essential documentation will be transferred to a third-party archiving service, which provides suitable fire and water-resistant facilities. Study files will be archived for a period of 10 years. Access to the study documentation is restricted to named individuals within the study team with express permission from the chief investigator.

### Statistical methods

All 37 patients who consented to and were recruited to the study will be included in the study flow diagram and the baseline characteristics tables. Descriptive statistics will be used to summarise the distribution of variables. Continuous variables will be reported with means and 95% CIs, if normally distributed, or medians and IQRs otherwise. Categorical variables will be reported using frequencies and percentages.

As the feasibility of the protocolised treatment regimen is core to the study design, a detailed investigation into the adherence of patients to the protocolised study treatment will be undertaken, including variation around the individual fractions’ dose and timing, as well as the received total dose and overall duration of each patient’s treatment course.

The analysis population for study endpoint assessment will include the set of patients who start treatment and provide information on treatment completion and any oesophagitis incidents, and their severity, occurring during their RT and for 4 weeks after completion. The primary outcome will be reported using a frequency, percentage and associated 95% CIs, and compared against the previously specified cut-off points for the stage 1 and stage 2 analyses. Weekly toxicity profiles will be reported using frequencies and percentages. To assess treatment efficacy, TTP from completion of RT will be graphically reported using a Kaplan-Meier plot, and rates and medians, with associated CIs, will be reported. PROMS on quality of life will be scored using appropriate manuals. No statistical testing will be performed. A detailed statistical analysis plan was drafted by the study statistician and approved by the chief investigator and an independent statistician prior to all data analysis timepoints.

### Data monitoring

The TMG meets on a monthly basis to discuss serious adverse events, quality of data entered, progress of the trial, protocol adherence, any proposed protocol amendments and any other problems.

### Interim analysis

The Simon’s two-stage design has been adopted for this study and analysis will be undertaken after 11 patients have primary endpoint information available, with the study being stopped early for futility if there are 5 or fewer patients who completed treatment and did NOT report grade 3–4 oesophagitis at this stage (ie, 6 or more who either failed to complete treatment, or reported grade 3–4 oesophagitis). Otherwise, recruitment will continue. This analysis will be performed by the study statistician based at Warwick Clinical Trials Unit and shared with the study’s oversight committee for the decision to be made regarding the continuation with stage 2.

### Trial monitoring

The trial co-ordinator/manager will have responsibility for overseeing day to day coordination of the study and reporting regularly to the TMG. The trial manager’s responsibilities include, but are not limited to:

- Coordinating protocol development, patient and study management documents.
- Correspondence with study funder and tracking of progress against agreed milestones.
- Setting up and maintaining the Master File.
- Ensuring necessary approvals are in place before the start of the study at each site.
- Providing training to study personnel.
- Providing data management support, including data input, maintenance of the study database and raising of queries.
- Producing study progress reports and coordinating TMG meetings and minutes.
- Ensuring data security and quality and ensuring data protection laws are adhered to.
- Ensuring complete records are in place for audit and monitoring purposes.
- Ensuring the study is conducted in accordance with the ICH GCP.
- Archiving all original study documents, including the data forms, in line with UHCW NHS Trust policy.

### Ancillary and post-trial care

After participation in the trial is completed at the 12-month visit (visit-9), the patient reverts to routine clinical follow-up and further management as required.

## Ethics and dissemination

### Research ethics approval

The study is conducted in compliance with the principles of the ICH GCP guidelines and in accordance with all applicable regulatory guidance, including, but not limited to, the UK policy framework for health and social care research. Health Research Authority and Health and Care Research Wales. Approval for this study was obtained on 18 April 2024, at the West Midlands—Solihull Research Ethics Committee (IRAS project ID: 332998; REC reference: 24/WM/0032). Progress reports and a final report at the conclusion of the study will be submitted to the approving REC within the timelines defined by the committee. Confirmation of capacity and capability was obtained from the R&D department prior to commencement of the study at all participating sites. The procedures are compliant with the Ionising Radiation (Medical Exposure) Regulations, and appropriate review by a Medical Physics Expert and Clinical Radiation Expert has been undertaken.

### Protocol amendments

The trial manager is responsible for communicating important protocol modifications to relevant parties.

### Informed consent

The chief investigator is responsible for taking informed consent from participants. Any other person that is delegated responsibility to participate in the informed consent process is duly authorised, trained and competent. The patient receives the PIS and consent form at the time of radiotherapy consent. Subsequently, the patient is expected to sign the consent a few days later, either at a subsequent clinic appointment or the visit for radiotherapy planning scan. The right of a participant to refuse participation without giving reasons is respected. Where new information is required to be provided to a participant or a participant is required to re-consent, the chief investigator is responsible for ensuring this is done in a timely manner.

The participant is free to withdraw at any time from the study without giving reasons and without prejudicing his/her further treatment. Data and samples collected up to the point of withdrawal will only be used after withdrawal if the participant has consented to this. Any intention to use such data is outlined in the consent literature.

If current developments or the availability of new information mean that continuing within the study is no longer ethical or in the best interests of a patient, that patient may need to be withdrawn from the study. Where any participant cannot read or write English and requires translators, appropriate alternative methods for supporting the informed consent process are employed. Vulnerable patients, such as those with cognitive impairment, are excluded from this feasibility study. The consent process involves:

- Discussion between the potential participant or his/her legally acceptable representative and an individual knowledgeable about the research about the nature and objectives of the study and possible risks associated with their participation.
- The presentation of written material (eg, information leaflet and consent form).
- The opportunity for potential participants to ask questions.
- Assessment of capacity. For consent to be ethical and valid in law, participants must be capable of giving consent for themselves.

### Data sharing

The source documentation includes electronic hospital records and treatment databases, including radiotherapy planning and treatment databases. The necessary data are collected and recorded onto a CRF. The CRF data will be used to perform statistical analysis for the study. As this is a multicentre study, we carry out direct electronic entry and automatic upload into the database, for speed efficiency and accuracy. The basic concept of source data is that it permits not only reporting and analysis but also verification at various steps in the process for the purposes of confirmation, quality control, audit or inspection. On confirmation of eligibility, participants are assigned a unique ID number, which is used to identify all documents associated with that participant for the duration of the study. All data, including source documents and eCRFs, is stored exclusively on hospital computer systems that are password-protected and access to these is defined and controlled by those on the delegation log.

### Harms

UHCW NHS Trust is acting as sponsor for this study and is undertaking the responsibilities of sponsor as defined by the UK Policy Framework for Health and Social Care Research and ICH GCP. An authorised representative of the Sponsor has approved the final version of this protocol with respect to the study design, conduct, data analysis and interpretation and plans for publication and dissemination of results. As sponsor, UHCW provides indemnity for this study and, as such, will be responsible for claims for any negligent harm suffered by anyone because of participating in this study.

Harm includes common side effects from thoracic RT. Online supplemental appendices 1–3 list the following information for management of common side effects:

- Online supplemental appendix 1: Common Terminology Criteria for Adverse Events: Version 4.03 (CTCAE_v4.03).
- Online supplemental appendix 2: Management of acute radiation-induced oesophagitis.
- Online supplemental appendix 3: Management of radiation-induced pneumonitis.

A copy of the radiotherapy guidelines with the radiotherapy dose constraints has also been included in online supplemental appendix 7.

### Confidentiality

The study complies with the current data protection regulations and regular checks and monitoring are undertaken by the trial co-ordinator to ensure compliance. Participants are assigned a unique identifier on enrolment into the study to allow link-anonymisation of patient-identifiable data. Access to patient identifiable data is restricted to members of the study co-ordination team who require it for the performance of their role. Electronic data are stored on password-protected encrypted drives and hard copies of study documents are stored in locked filing cabinets in secure entry-card protected sites.

### Dissemination policy

Once completed, the study results will be sent to participants in a plain language summary and published in a peer-reviewed journal. For updates, please visit (https://research.mededcoventry.org/About-Us/Meet-The-Team/TMU/Ship-Rt).

### Trial status

The study is currently running under protocol version 1.2 (dated 19 November 2025). Recruitment started in November 2024, and the study remains on track for completion in March 2027. To date, we have recruited 23 patients,^27^ and the stage 1 analysis concluded that study recruitment should continue to the full 37 patient target. For updates on the study, please visit the study web page (https://research.mededcoventry.org/About-Us/Meet-The-Team/TMU/Ship-Rt).

## Supplementary material

10.1136/bmjopen-2025-111350 online supplemental file 1

10.1136/bmjopen-2025-111350 online supplemental file 2
