# Supplementary material for: Shortened High-dose Palliative Radiotherapy for Lung Cancer (SHiP-Rt): protocol for a single-arm, multicentre, phase II study
Source: BMJ Open. 2026 Feb 2;16(2):e111350. doi: 10.1136/bmjopen-2025-111350 (PMC12878348; doi:10.1136/bmjopen-2025-111350)

Appendix 1 – Common Terminology Criteria for Adverse Events: Version 4.03 (CTCAE_v4.03)

Appendix 2 – Management of acute radiation induced oesophagitis

Appendix 3 – Management of radiation induced pneumonitis

Appendix 4 – ECOG Performance Status

Appendix 5 – Participant Information Sheet

Appendix 6 – Trial Consent Form

Appendix 7 – Radiotherapy planning and delivery guidelines

**Appendix 1**

Common Terminology Criteria for Adverse Events (CTCAE) Common Terminology Criteria for Adverse Events: Version 4.03 (CTCAE_v4.03) Publish Date: June 14, 2010.

Accessed at: [Common Terminology Criteria for Adverse Events (CTCAE)](https://evs.nci.nih.gov/ftp1/CTCAE/CTCAE_4.03/CTCAE_4.03_2010-06-14_QuickReference_8.5x11.pdf)

**Appendix 2: Management of Acute Radiation Induced Oesophagitis**

Inflammation of the thoracic oesophagus is a common acute side effect of lung cancer radiotherapy. Changes in radiotherapy dose-fractionation can increase the severity of acute radiation oesophagitis. The management principles of acute oesophagitis are outlined below.

The symptoms of oesophagitis are dysphagia and pain. Patients may experience pain in the retrosternal region, back or epigastrium. Moderate dysphagia may necessitate a change to a soft or liquid diet. In severe cases patients may require fine bore nasogastric feeding. Time Course Symptoms of oesophagitis may begin in the second week of a course of radiotherapy and can increase progressively thereafter. The symptoms may continue to worsen for one or two weeks after the course of radiation is completed, and then gradually settle over a period of four to six weeks. If oesophagitis has not started to settle by 6-8 weeks, then chronic oesophagitis should be suspected, and expert gastroenterological assessment sought.

Radiation oesophagitis typically occurs if part of the oesophagus lies within the PTV. The degree of oesophagitis and severity of symptoms is likely to increase with the dose delivered and the length of oesophagus included within the PTV. Even if the oesophagus is not included within the PTV, symptomatic oesophagitis may occur if any oesophageal mucosa receives a radiation dose equal to or exceeding 20 Gy over the treatment course.

Patients should be warned of the possibility of oesophagitis, with particular reference to the clinical features and management options, before starting treatment

Management

Particular care is required to ensure that the patient maintains an adequate fluid intake throughout treatment and afterwards. Weekly on-treatment assessment is essential during and up to 4 weeks after radiotherapy within this study. Some patients may require referral to a dietician, to help control symptoms and ensure that nutritional intake is appropriate and dietary supplements are introduced at the appropriate time. Radiation esophagitis should be self-limiting, but early symptomatic control is important to help patients maintain their fluid and nutritional intake. The following medications can be helpful in this respect:

1. Analgesics

The standard approach in radiation oesophagitis is to introduce regular mild analgesics at the onset of symptoms and escalate analgesic potency as severity of symptoms increases. Dispersible, soluble, or liquid forms of oral medication should be used whenever possible.

• Soluble Paracetamol: 1g every 4-6 hours (maximum 4g in 24 hours)

• Dispersible Paracetamol and Codeine: Paracetamol 1g with Codeine every 4-6 hours (maximum 4g of paracetamol in 24 hours). Caution is required as all opiate analgesics can cause constipation.

• Strong Opiate Analgesics: Caution is required as all opiate analgesics can cause constipation. o Liquid Morphine: Initially 5-10 mg 4 hourly; Escalate as required to ensure pain relief. o Oxycodone: Patients with opiate sensitive pain but are unable to tolerate morphine, oxycodone may be substituted. 1mg of oxycodone is equivalent to 2 mg of oral morphine.

1. Local Anaesthetics

Many patients report benefit from swallowing a local anaesthetic preparation before meals. This should be used in conjunction with the analgesic ladder described above.

• Oxceticaine and antacid combination: 10ml taken 15 minutes before meals.

1. Proton Pump Inhibitors

Inflammation of the lower oesophagus may reduce the efficacy of the oesophago- gastric sphincter mechanism. Any reflux of acid into the inflamed oesophagus will exacerbate the symptoms of radiation oesophagitis. Therefore, a proton pump inhibitor (PPI) should be used if part of the mid or lower third of the oesophagus is included in the planning target volume. Patients with a hiatus hernia should also receive a PPI.

• Lansoprazole, 30 mg po once daily

• Omeprazole, 20 mg po once daily

4. Steroids

For moderate to severe oesophagitis, steroids can be useful to reduce inflammation and ameliorate dysphagia. A PPI should be given routinely to patients taking steroids.

• Dexamethasone, 4-8 mg suspension daily.

1. Oesophageal Candidiasis

Clinicians should be aware that radiation oesophagitis can predispose patients to superadded infection with candida albicans; this is a particular risk if the patient is on steroids or is diabetic.

• Fluconazole, 100mg suspension daily for 5 days 7. Other Approaches Feeding and hydration via fine bore nasogastric tube: Normal tissue reactions to radiation can vary in severity for some patients. If a patient starts to develop dysphagia in the first week of radiotherapy, or if symptoms of radiation oesophagitis are unusually severe in week two, consideration should be given to early insertion of a fine bore nasogastric tube to ensure continuation of enteral hydration and nutrition.

Follow Up

After completion of radiotherapy, patients with radiation oesophagitis should be reviewed weekly until the symptoms of oesophagitis start to resolve. Usually, the symptoms will be markedly improved by around four weeks after completion of radiotherapy and should be much better by 6-8 weeks after completion of treatment. If patients are still having difficulty swallowing solids eight weeks after treatment has finished, referral for endoscopy is indicated

**Appendix 3: Management of Radiation Induced Pneumonitis**

Radiation Pneumonitis is far less commonly seen or described in patients receiving high dose palliative RT. This can be divided into early pneumonitis, occurring between one and four months after completion of radiotherapy, and late pneumonitis, which may cause symptoms six months to two years after treatment and is a permanent change.

Early radiation pneumonitis (ERTP) occurs due to inflammatory changes within the lung tissue. Patients may develop a cough, possibly productive of clear or white sputum, and breathlessness. A CXR may show patchy inflammatory changes or consolidation within the irradiated lung volume. An uncommon variant of ERTP is Bronchiolitis Obliterans with Organising Pneumonia (BOOP). This usually involves lung tissue outside the radiation volume. Fever and cough are usually the predominant symptoms. CXR shows inflammatory infiltrates which can be both remote from and within the irradiated area. Patients respond dramatically to steroid therapy.

Late radiation pneumonitis (LRTP) comprises scarring and fibrosis of lung tissue and is a permanent change. The symptoms are breathlessness and dry cough. CXR and CT scanning show loss of lung volume in the affected area and fibrotic changes.

Major problems with radiation pneumonitis from lung cancer radiotherapy can usually be avoided by careful patient selection and radiotherapy technique. The use of V20 recognises that any lung tissue receiving 20 Gy or more will undergo fibrosis. Therefore, at any level of V20 the clinician should consider whether the individual patient will be able to cope without the percentage of functioning lung tissue indicated. Note that V20 is defined as the total volume of lung tissue minus the GTV, that receives 20 Gy or more. V20 is rarely an issue in patients receiving high dose palliative RT.

Treatment

Management of acute and chronic respiratory complications is outlined below. Smoking can exacerbate the detrimental effects of both early and late radiation pneumonitis and any patients who are still smoking should be encouraged to stop.

ERTP: Early treatment with steroids (e.g., dexamethasone 6-8 mg daily for one week depending on severity, reducing to 4 mg daily for a further week or more, and then tailing off gradually; or 1mg/kg prednisolone daily for significant symptoms, 0.5mg/kg for less severe symptoms) will usually produce symptomatic improvement. A concomitant respiratory tract infection should be looked for and treated, if found. If there is no symptomatic improvement with steroids and antibiotics, consideration should be given to the possibilities of pulmonary emboli, cardiac failure and tumour progression.

LRTP: As LRTP is due to permanent changes in the lung tissue there is no restorative treatment. Therapeutic effort is concentrated upon reducing the degree of respiratory compromise and avoiding or minimising the effect of recurrent respiratory tract infections. Some patients may benefit from long courses of low to intermediate dose steroid therapy (e.g., 5-20 mg of prednisolone per day) and in extreme cases oxygen therapy may be required. Referral to respiratory physician is recommended for opinion and input.

**Appendix 4: ECOG Performance Status**


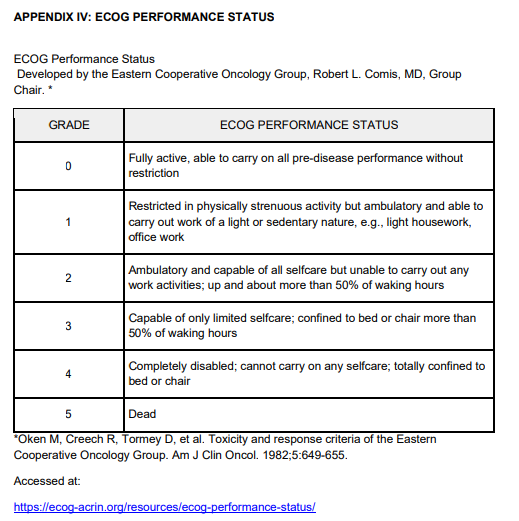


**Appendix 5 – Participant Information Sheet**


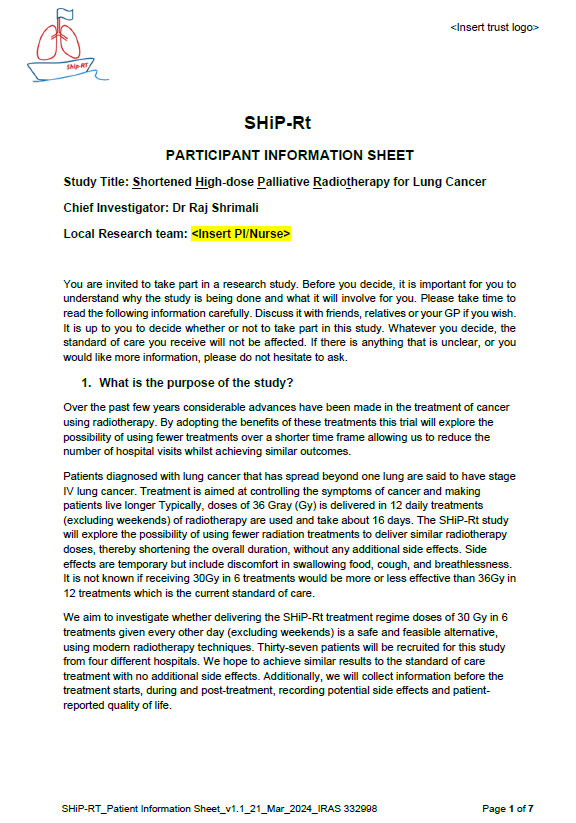


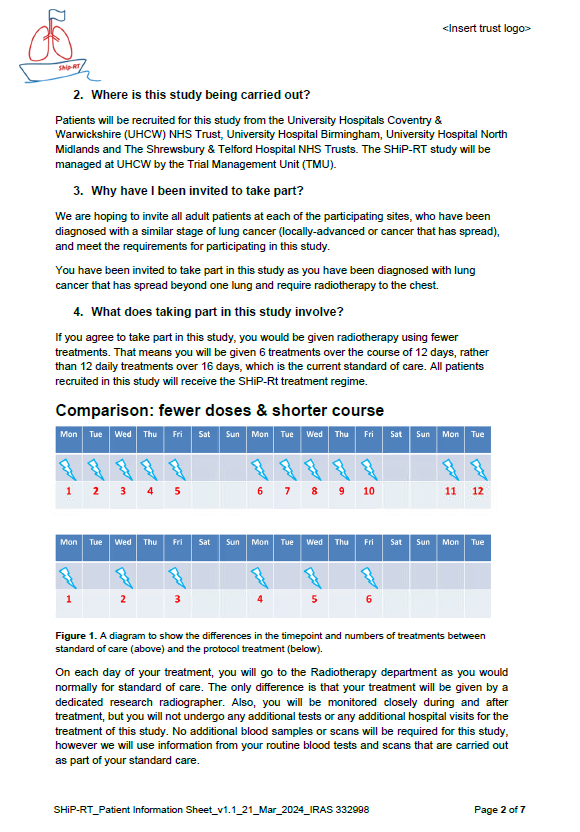


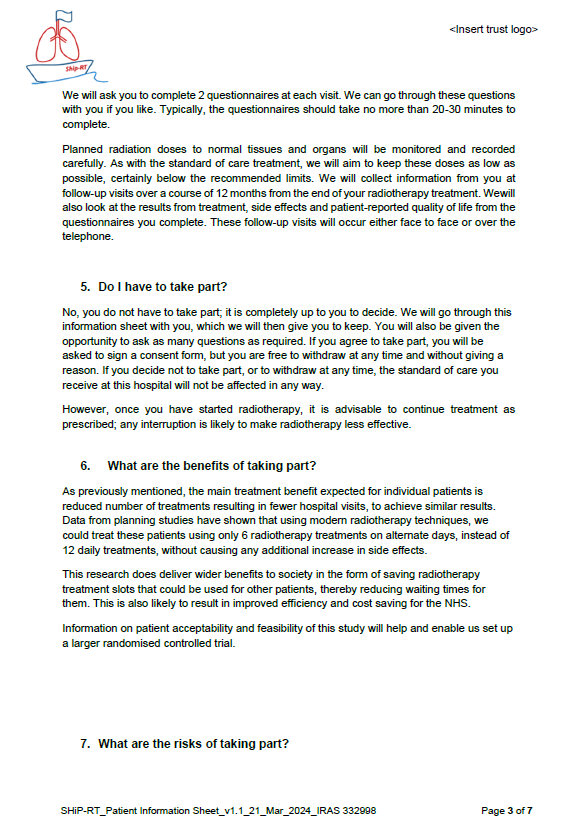


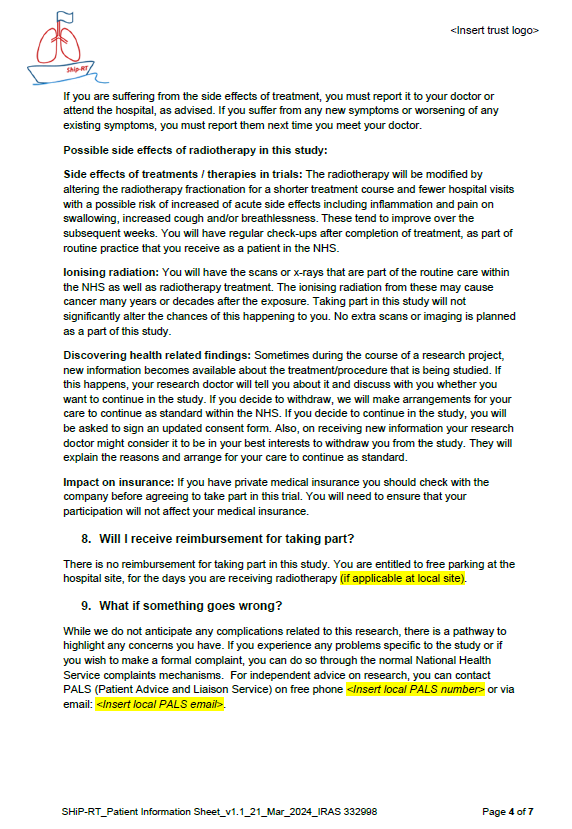


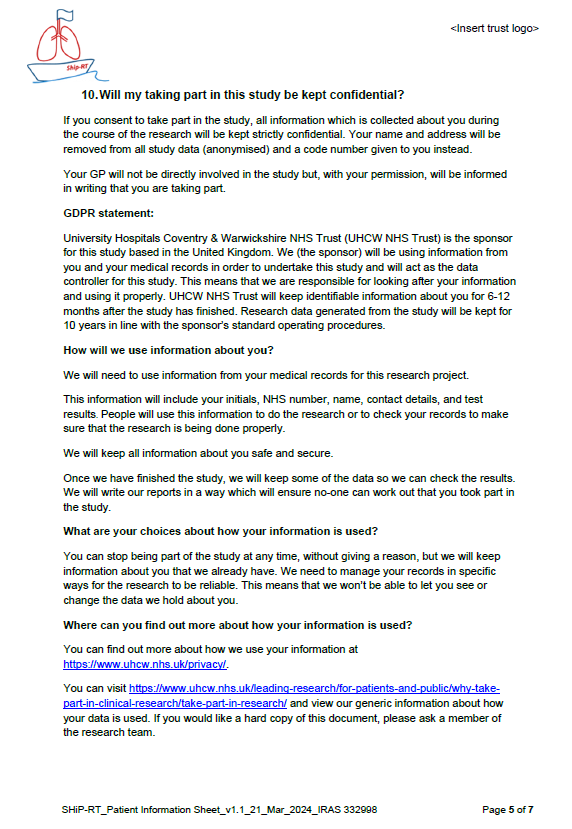


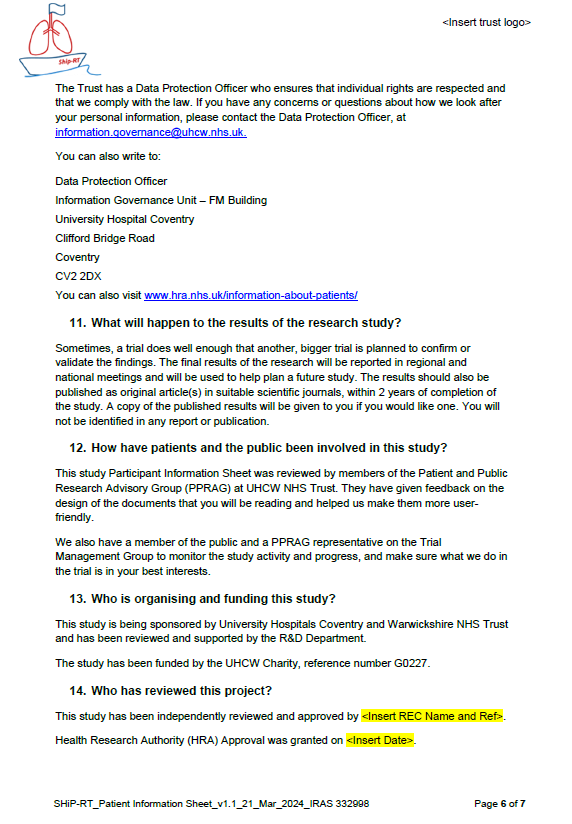


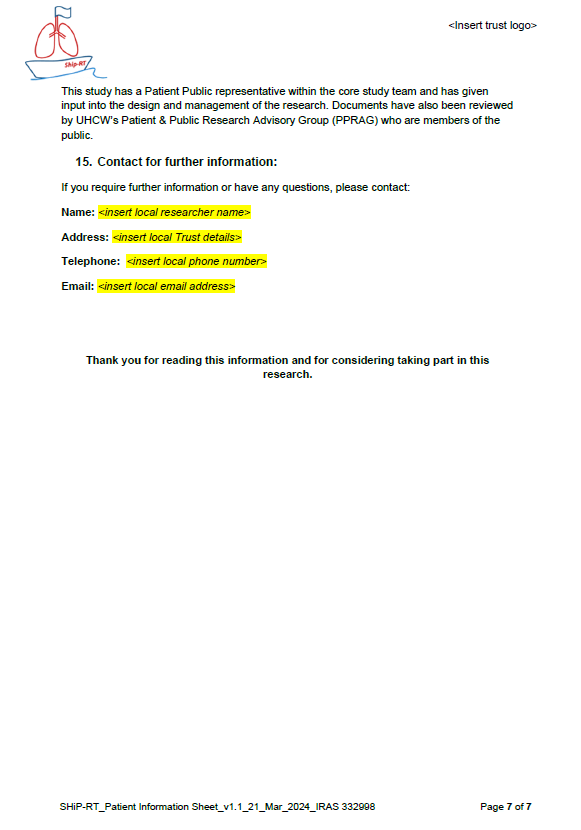


**Appendix 6 – Trial Consent Form**

**
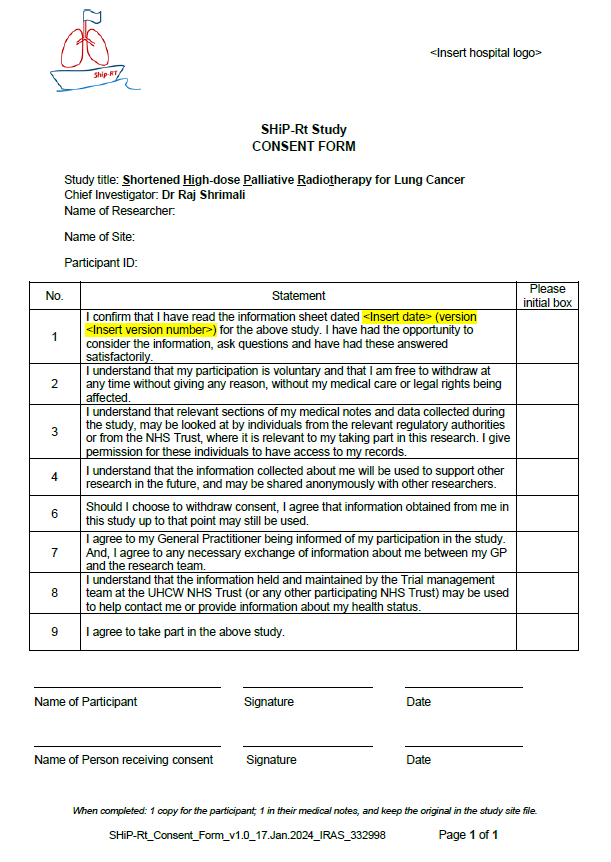
**

**Appendix 7 – Radiotherapy planning and delivery guidelines**


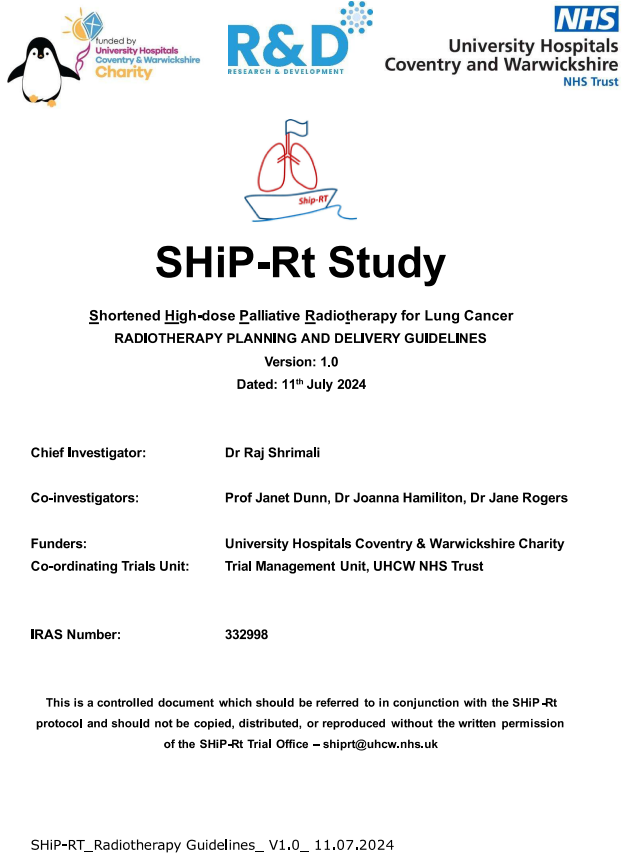


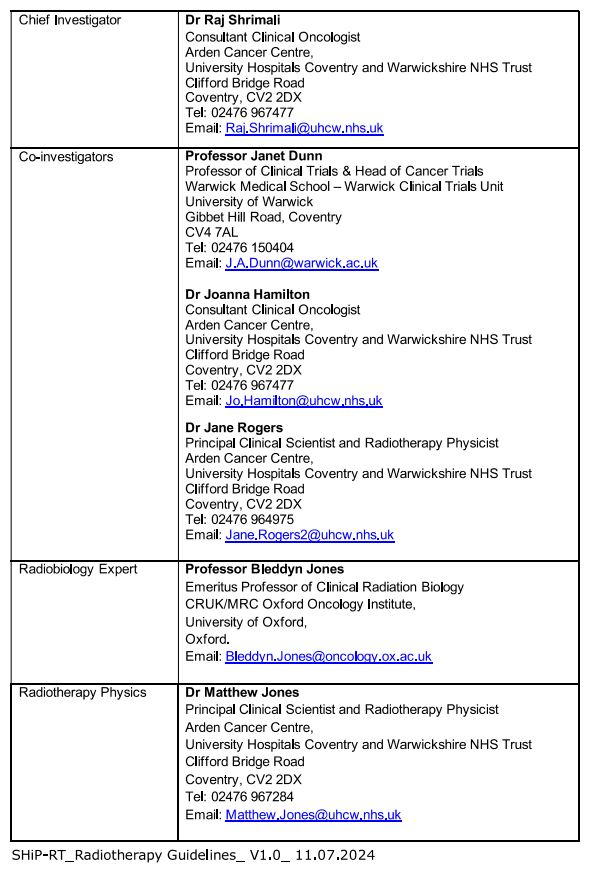


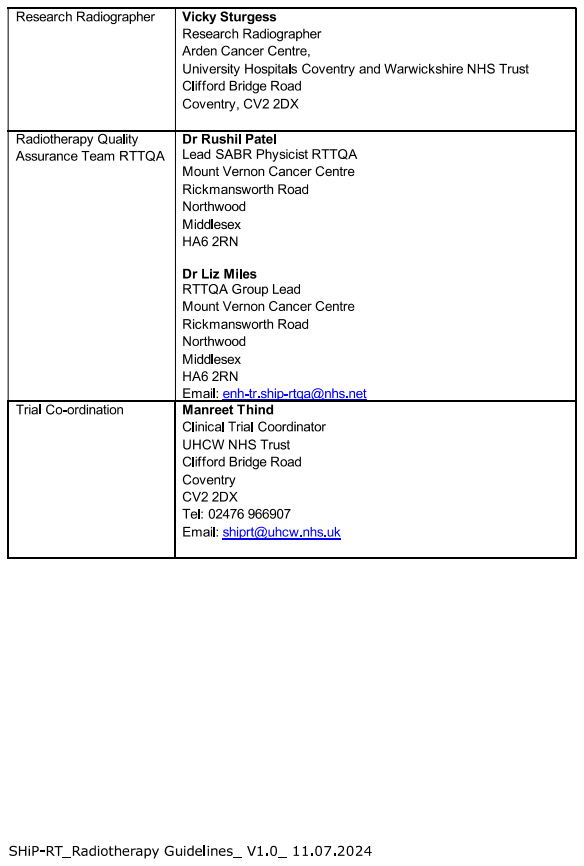


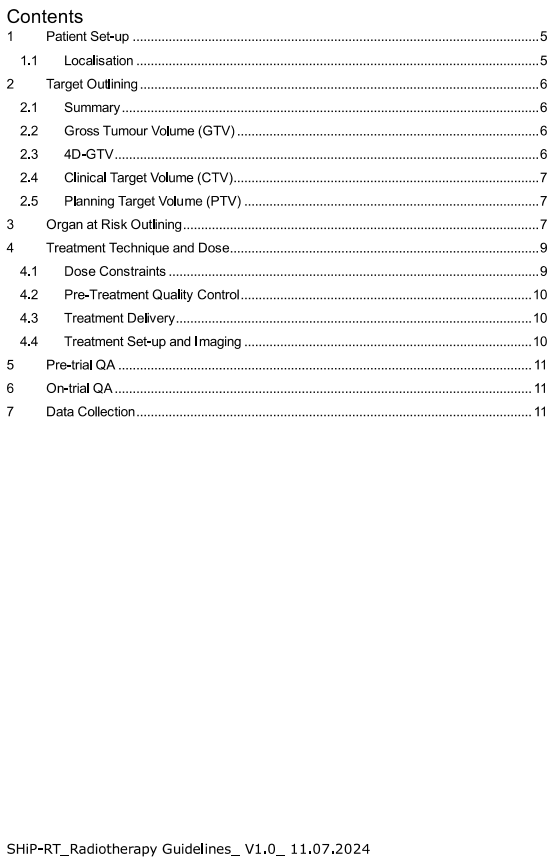

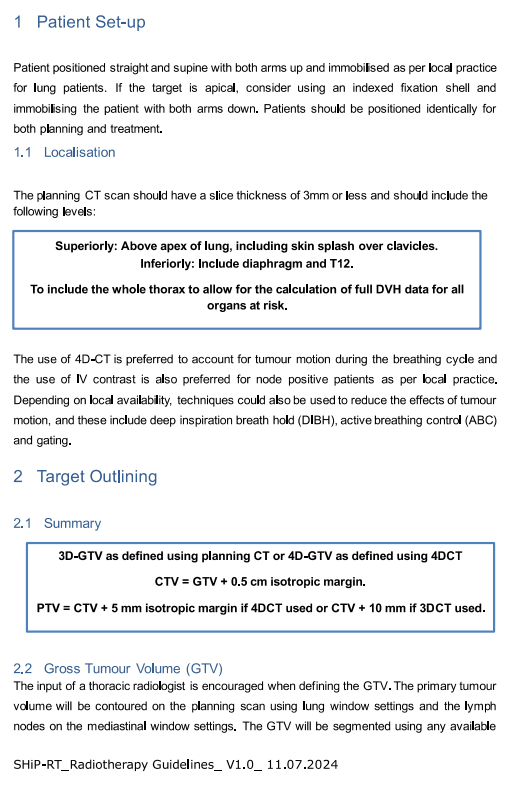


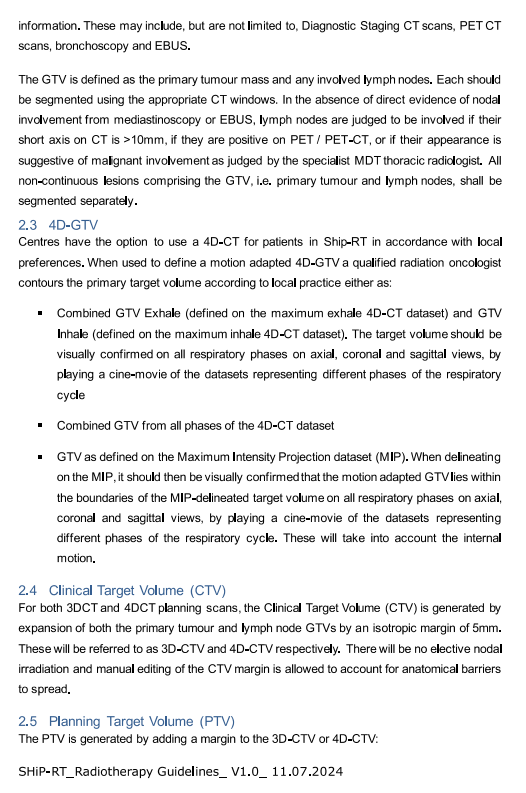

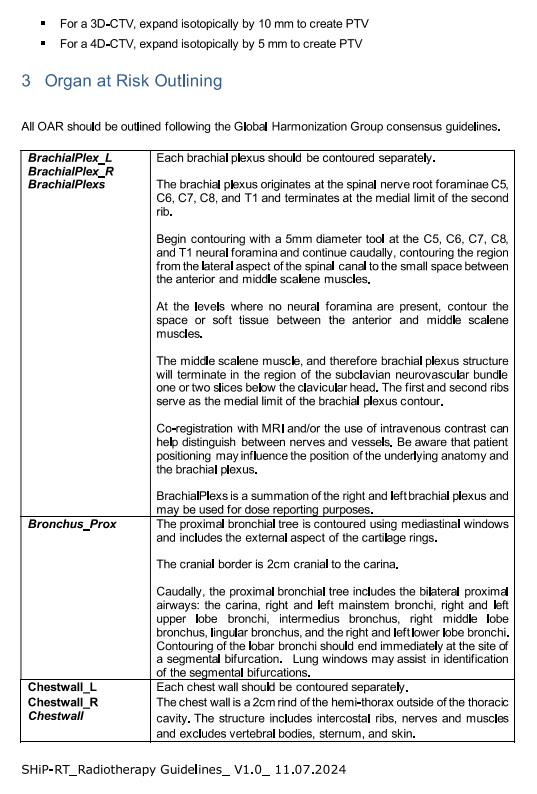


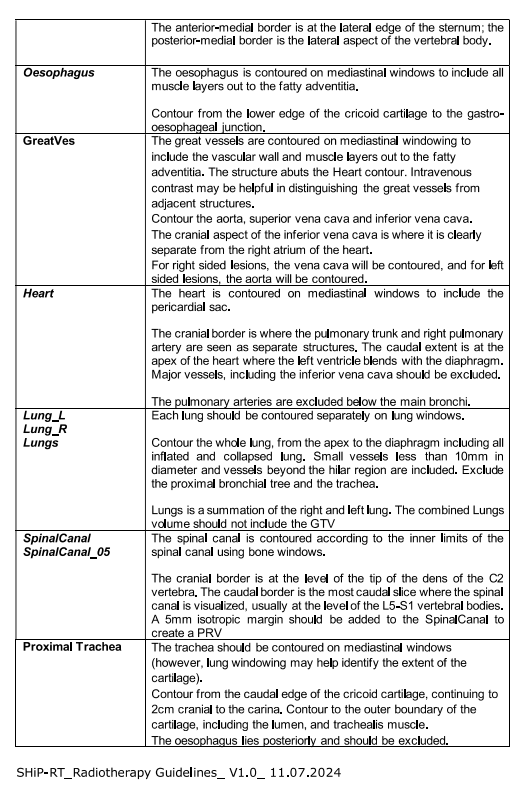

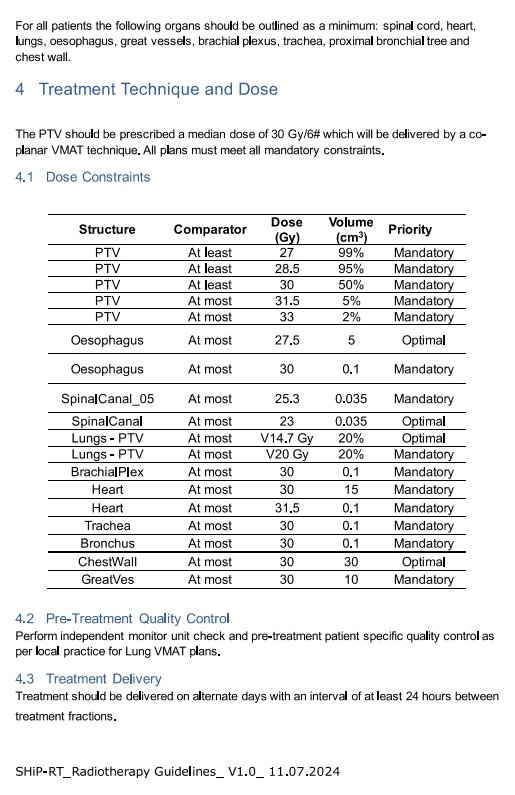


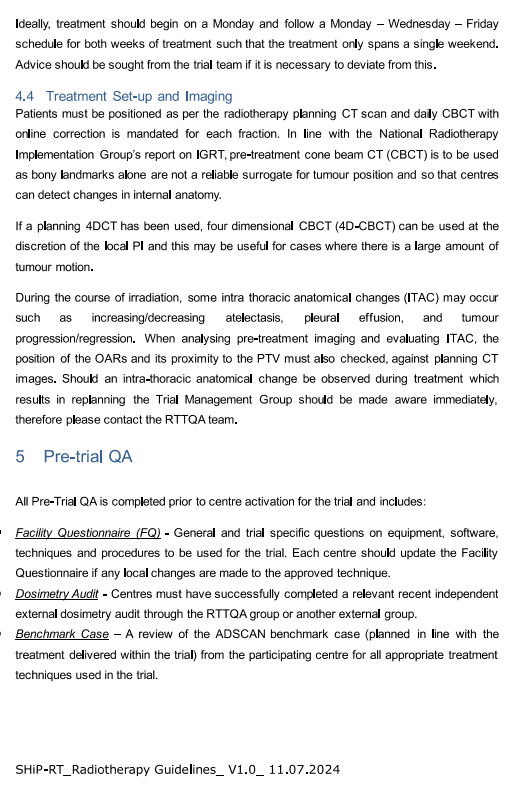


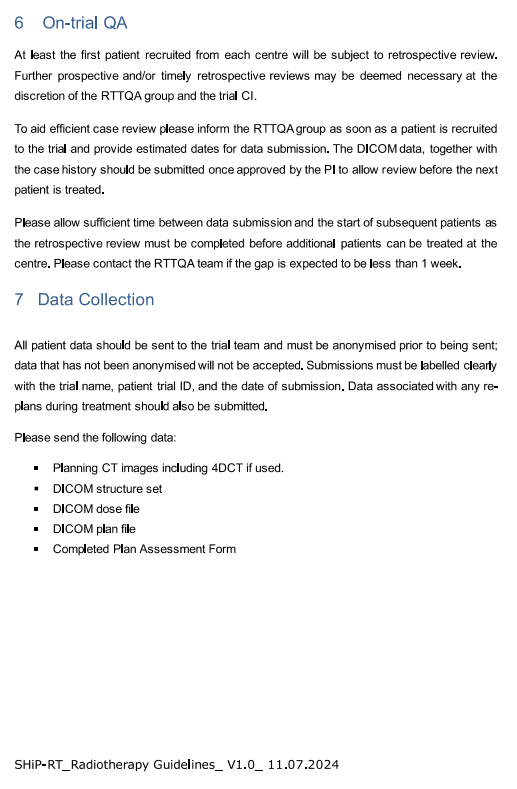

Supplement: online supplemental file 1 [file bmjopen-16-2-s001.docx]
