# Supplementary material for: Shortened High-dose Palliative Radiotherapy for Lung Cancer (SHiP-Rt): protocol for a single-arm, multicentre, phase II study
Source: BMJ Open. 2026 Feb 2;16(2):e111350. doi: 10.1136/bmjopen-2025-111350 (PMC12878348; doi:10.1136/bmjopen-2025-111350)
Supplement: online supplemental file 2 [file bmjopen-16-2-s002.docx]

**Roles and Responsibilities**

| Chief Investigator | Dr Raj K Shrimali  Consultant Clinical Oncologist  Arden Cancer Centre,  University Hospitals Coventry and Warwickshire NHS Trust  Clifford Bridge Road  Coventry, CV2 2DX  Tel: 02476 967477  Email: Raj.Shrimali@uhcw.nhs.uk |
| --- | --- |
| Co-investigator | Professor Janet Dunn  Professor of Clinical Trials & Head of Cancer Trials  Warwick Medical School – Warwick Clinical Trials Unit  University of Warwick  Gibbet Hill Road, Coventry  CV4 7AL  Tel: 02476 575847  Email: J.A.Dunn@warwick.ac.uk |
| Co-investigator | Dr Joanna Hamilton  Consultant Clinical Oncologist  Arden Cancer Centre,  University Hospitals Coventry and Warwickshire NHS Trust  Clifford Bridge Road  Coventry, CV2 2DX  Tel: 02476 967477  Email: Jo.Hamilton@uhcw.nhs.uk |
| Co-investigator | Dr Jane Rogers  Principal Clinical Scientist and Radiotherapy Physicist  Arden Cancer Centre,  University Hospitals Coventry and Warwickshire NHS Trust  Clifford Bridge Road  Coventry, CV2 2DX  Tel: 02476 964975  Email: Jane.Rogers2@uhcw.nhs.uk |
| Sponsor | Miss Sonia Kandola  University Hospitals Coventry and Warwickshire NHS Trust  Clifford Bridge Road  Coventry, CV2 2DX  Tel: 02476 965031  Email: ResearchSponsorship@uhcw.nhs.uk |
| Funder | Mr. David Lloyd, Charity Grants Administrator  University Hospitals Coventry & Warwickshire Charity  Main Reception, UHCW NHS Trust  Clifford Bridge Road  Coventry, CV2 2DX  Tel: 02476 9669059  Email: David.Lloyd@uhcw.nhs.uk |
| Statistician | Dr Louise Hiller  Associate Professor  Warwick Medical School – Warwick Clinical Trials Unit  University of Warwick  Gibbet Hill Road, Coventry  CV4 7AL  Tel: 02476150179  Email: L.Hiller@warwick.ac.uk |
| Radiobiology Expert | Professor Bleddyn Jones  Emeritus Professor of Clinical Radiation Biology  CRUK/MRC Oxford Oncology Institute,  University of Oxford,  Oxford.  Email: Bleddyn.Jones@oncology.ox.ac.uk |
| Radiotherapy Physics | Dr Matthew Jones  Principal Clinical Scientist and Radiotherapy Physicist  Arden Cancer Centre,  University Hospitals Coventry and Warwickshire NHS Trust  Clifford Bridge Road  Coventry, CV2 2DX  Tel: 02476 967284  Email: Matthew.Jones@uhcw.nhs.uk |
| Trial Co-ordination | Manreet Thind  Clinical Trial Coordinator  UHCW NHS Trust  Clifford Bridge Road  Coventry  CV2 2DX  Tel: 02476 966907  Email: shiprtstudyinbox@uhcw.nhs.uk |
| Principle Investigators | 1. Dr Apurna Jegannathen, Consultant Clinical Oncologist,  University Hospitals of North Midlands NHS Trust, Stoke  on Trent.  2. Dr Qamar Ghafoor, Consultant Clinical Oncologist,  University Hospitals Birmingham NHS Foundation Trust,  Birmingham.  3. Dr Anirban Chatterjee, Consultant Clinical Oncologist,  The Shrewsbury and Telford Hospital NHS Trust,  Shrewsbury. |
| PPRAG Representative | Mr Charles Peebles  Lay member and Patient and Public Research Advisory Group  (PPRAG) representative, UHCW NHS Trust, Coventry, CV2 2DX. |
