# Supplementary material for: Shortened High-dose Palliative Radiotherapy for Lung Cancer (SHiP-Rt): protocol for a single-arm, multicentre, phase II study
Source: BMJ Open. 2026 Feb 2;16(2):e111350. doi: 10.1136/bmjopen-2025-111350 (PMC12878348; doi:10.1136/bmjopen-2025-111350)
Supplement: Supplementary file 3 [file bmjopen-2025-111350.reviewer_comments.pdf]

PEER REVIEW HISTORY

BMJ Open publishes all reviews undertaken for accepted manuscripts. Reviewers are asked to complete a checklist review form and are provided with free text boxes to elaborate on their assessment. These free text comments are reproduced below.

ARTICLE DETAILS

Title (Provisional)

Shortened High-dose Palliative Radiotherapy for lung cancer (SHiP-Rt): protocol for a single-arm, multicentre, phase II study

Authors

Shrimali, Raj Kumar; Griffin, Ellanna; Sturgess, Vicky; Jones, Matthew; Hiller, Louise; Rogers, Jane; Hamilton, Joanna; Peebles, Charles; Jones, Bleddyn; Dunn, Janet

---

VERSION 1 - REVIEW

**Reviewer**	**1**
**Name**	**Williams, Norman**
**Affiliation**	**University College London, UCL Division of Surgery & Interventional Science**
**Date**	**17-Dec-2025**
**COI**	**None**

The manuscript describes a protocol that addresses an important clinical question: whether contemporary radiotherapy techniques can safely deliver high-dose palliative RT in fewer fractions for people with metastatic NSCLC. Making use of modern VMAT and image-guided techniques to shorten treatment duration could improve patient experience and have economic benefits for the health service.

The manuscript is very well-written. However, there are some concerns that need to be addressed before a decision can be made to proceed to publication.

There seems to be a lack of consistency of the wording of the primary outcome, which is essentially a composite of protocol adherence (completion of the radiation schedule) and development of a particular side-effect (oesophagitis). The Abstract states "The SHiP-Rt study aims to evaluate the safety and efficacy of reducing the number of radiotherapy (RT) fractions and RT duration..." (page 3 line 9), but isn't the term "efficacy" misleading? Particularly as the Introduction (page 6 line 27) states "Treatment for stage-IV NSCLC is

aimed at maintaining or improving quality of life." Similarly, the Abstract Conclusion (page 3 line 29) states "If successful, this study will support a phase-III randomised controlled trial to assess efficacy."

The use of Simon’s two-stage design is appropriate for this type of study. However, it is not clear when the Stage 1 assessment will take (or has taken) place within the timeframe of the study. It is stated (page 13 line 20) "Stage-1 analysis: Patients will be accrued until 11 patients have primary endpoint information available. If there are 5 or fewer patients who completed treatment and did NOT report grade 3-4 oesophagitis ..." The RT takes two weeks, and the oesophagitis assessment is made four weeks after that, so six weeks in total after the 11th recruited participant. According to [1], "The effectiveness of shorter radiotherapy treatments in improving the quality of life for terminally ill lung cancer patients is being explored by University Hospitals Coventry and Warwickshire (UHCW) NHS Trust....The first 23 of 37 patients have now been recruited..." Does this mean that Stage 1 was "successful" and the sample size will be 37?

Page 8 line 51: Spell out the symbol #

Page 13 line 16 states a sample size of 37 whereas line 26 implies a sample size of 31 - please clarify.

Page 14 line 16 states "A detailed statistical analysis plan (SAP) will be drafted by the study statistician,

which will be finalised and approved by the CI and an independent statistician before the final data

analysis." Shouldn't the SAP be finalised before the first of Simon's Two Step?

References

[1] https://www.uhcw.nhs.uk/news/shorter-radiotherapy-treatments-could-improve-quality-of-life-for-patients-with-advanced-lung-cancer/ (accessed on 11 December 2025)

VERSION 1 - AUTHOR RESPONSE

Response to Reviewer 1

Dear Professor Williams,

Many thanks for your thoughtful and constructive comments; they are very much appreciated. I would like to discuss the changes we have made in response to your feedback.

Point 1: There seems to be a lack of consistency of the wording of the primary outcome, which is essentially a composite of protocol adherence (completion of the radiation schedule) and development of a particular side-effect (oesophagitis). The Abstract states "The SHiP-Rt study aims to evaluate the safety and efficacy of reducing the number of radiotherapy (RT) fractions and RT duration..." (page 3 line 9), but isn't the term "efficacy" misleading? Particularly as the Introduction (page 6 line 27) states "Treatment for stage-IV NSCLC is aimed at maintaining or improving quality of life." Similarly, the Abstract Conclusion (page 3 line 29) states "If successful, this study will support a phase-III randomised controlled trial to assess efficacy."

Response to point 1: We agree that there was some inconsistency in the wording of the primary outcome and we have revised this to clearly reflect that this Phase 2 study focuses on safety and feasibility. Should this phase be successful, a subsequent Phase 3 study will assess efficacy.

Point 2: The use of Simon's two-stage design is appropriate for this type of study. However, it is not clear when the Stage 1 assessment will take (or has taken) place within the timeframe of the study. It is stated (page 13 line 20) "Stage-1 analysis: Patients will be accrued until 11 patients have primary endpoint information available. If there are 5 or fewer patients who completed treatment and did NOT report grade 3-4 oesophagitis ..." The RT takes two weeks, and the oesophagitis assessment is made four weeks after that, so six weeks in total after the 11th recruited participant. According to [1], "The effectiveness of shorter radiotherapy treatments in improving the quality of life for terminally ill lung cancer patients is being explored by University Hospitals Coventry and Warwickshire (UHCW) NHS Trust....The first 23 of 37 patients have now been recruited..." Does this mean that Stage 1 was "successful" and the sample size will be 37?
Point 4: Page 13 line 16 states a sample size of 37 whereas line 26 implies a sample size of 31 - please clarify.

Response to points 2 & 4: Thank you for clarifying the issues surrounding the Simon two-stage design. We have added a statement to clarify that the total sample size is 37 patients, allowing for a 15% attrition rate. You are correct that Stage 1 has been successfully completed, as demonstrated by the article you identified, and we agree that referencing this within the protocol improves clarity.

Point 3: Page 8 line 51: Spell out the symbol #

Response to point 3: Thank you for identifying this error; the symbol has now been replaced by the word, "fraction(s)," and the abbreviation has been removed.

Point 5: Page 14 line 16 states "A detailed statistical analysis plan (SAP) will be drafted by the study statistician, which will be finalised and approved by the CI and an independent statistician before the final data analysis." Shouldn't the SAP be finalised before the first of Simon's Two Step?

Response to point 5: Statistical plan has now been clarified and included. We have also clarified that the detailed statistical analysis plan (SAP) was drafted and approved prior to all data analysis timepoints.

VERSION 2 - REVIEW

Reviewer 1

Name Williams, Norman

Affiliation University College London, UCL Division of Surgery & Interventional Science

Date 09-Jan-2026

COI

The authors have addressed all comments satisfactorily and the manuscript can now be recommended for acceptance.
